# It’s a kind of MAGIC: uncovering the origins of chromosomal instability

**DOI:** 10.1038/s41392-026-02588-6

**Published:** 2026-02-05

**Authors:** David Gómez-Peregrina, César Serrano

**Affiliations:** 1 Sarcoma Translational Research Laboratory, Vall d’Hebron Institute of Oncology (VHIO), Barcelona, Spain; 2 Department of Medical Oncology, Vall d’Hebron University Hospital, Barcelona, Spain

**Keywords:** Cell biology, Molecular biology

In a recent study published in Nature, Cosenza et al. ^1^ introduced MAGIC (machine-learning-assisted genomics and imaging convergence), a versatile platform that photolabels cells with micronuclei and other nuclear atypia, enabling direct quantification of de novo chromosomal abnormality (CA) formation and ongoing chromosomal instability (CIN) at single-cell resolution. Using this framework, the authors show how spontaneous and induced lesions from pre-mitotic DNA damage and mitotic errors drive distinct CIN trajectories, with *TP53* inactivation markedly amplifying CA burden and complexity, highlighting how CIN emergence and propagation prior to clonal expansion may inform tumour initiation, progression and the identification of actionable biomarkers and therapeutic vulnerabilities.

**Fig. 1:**
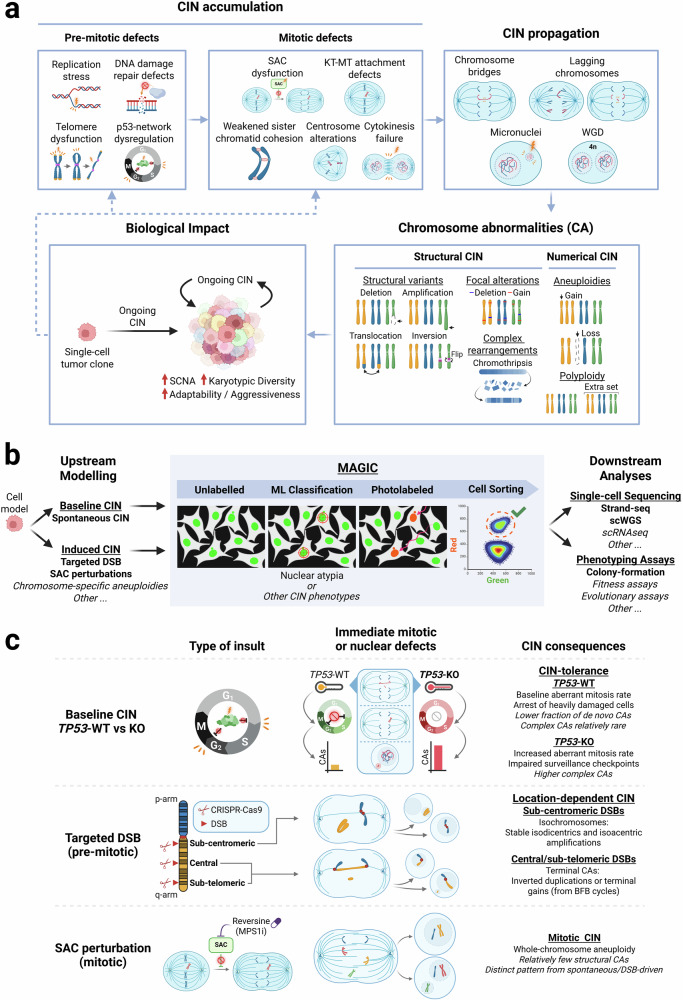
MAGIC as a framework to dissect the origins and consequences of chromosomal instability. **a** Ongoing CIN and biological impact. Pre-mitotic defects (for example, replication stress, DNA repair defects, telomere dysfunction and p53 network dysregulation) and mitotic errors (for example, spindle assembly checkpoint (SAC) dysfunction, kinetochore–microtubule attachment errors, cohesion defects, centrosome abnormalities and cytokinesis failure) give rise to chromosome bridges, lagging chromosomes, micronuclei and whole-genome doubling (WGD). These processes drive ongoing CIN, increasing karyotypic diversity through numerical and structural CAs (whole-chromosome aneuploidies, polyploidy, focal copy number changes and complex rearrangements) and reshaping tumour phenotypes. **b** MAGIC workflow and experimental space. Upstream modelling (left) encompasses baseline CIN and induced CIN modelling (for example, targeted DSBs, SAC perturbations and chromosome-specific aneuploidies), which are coupled to the MAGIC pipeline (centre) consisting of high-content live-cell imaging, machine-learning-based detection of nuclear atypia, photolabelling and FACS isolation. Downstream analyses (right) include single-cell sequencing (Strand-seq, scWGS, scRNA-seq) and phenotyping assays. Elements in bold indicate perturbations and readouts implemented by Cosenza et al. ^1^; elements in italics indicate additional strategies that could, in principle, be coupled to MAGIC. **c** MAGIC readouts for different CIN origins. Top, baseline CIN (spontaneous CAs) in *TP53*-wild-type (*TP53*-WT) versus *TP53*-knockout (*TP53*-KO) cells and their distinct CIN tolerance regimens. Middle, pre-mitotic targeted DSBs at sub-centromeric, central or sub-telomeric positions generate characteristic immediate defects (centric bridges, lagging acentrics, micronuclei) and location-dependent CIN, including dicentric and acentric isochromosomes, terminal CAs and inverted duplications from breakage-fusion-bridge (BFB) cycles. Bottom, mitotic CIN induced by MPS1 inhibition (reversine) produces whole-chromosome aneuploidy with relatively few structural CAs, yielding a pattern distinct from spontaneous or DSB-driven CIN

CIN is the ongoing process by which cells acquire new CAs through repeated chromosome missegregation and structural rearrangements, altering chromosome number and structure across divisions (Fig. 1a). CAs are pervasive across cancers and typically affect more genes than substitutions or small insertion/deletion alterations, fuelling clonal diversification and tumour adaptation.^2–5^ Accurate chromosome segregation and genome integrity rely on coordinated processes such as DNA replication and repair, telomere maintenance, spindle assembly and mitotic exit.^2^ When these are compromised and cells bypass senescence and apoptosis, concurrent pre-mitotic and mitotic defects drive ongoing CIN.^2,5^ However, most CIN knowledge still comes from bulk copy-number landscapes in tumours rather than direct measurements of distinct CIN processes and how they shape these patterns, a gap that MAGIC begins to close.^1,3^

To capture CIN as it unfolds, MAGIC autonomously scans thousands of cells, uses a machine-learning classifier to detect nuclear atypia, photoconverts fluorescent labels in selected cells by targeted laser excitation and isolates them by FACS for single-cell sequencing and phenotyping (Fig. 1b). MAGIC is then applied to two near-diploid, non-transformed epithelial models, MCF10A and hTERT-RPE-1, engineered to express H2B-Dendra2 or a photoactivatable dye for chromatin labelling. Baseline CIN is addressed with the identification of spontaneous nuclear atypia, including micronuclei, while CIN induction is modelled using CRISPR-Cas9-induced double-strand breaks (DSBs) and transient MPS1 inhibition with reversine (Fig. 1b, c). By integrating nuclear morphology, lineage relationships between sister cells, genome structure and biological phenotypes, MAGIC reconstructs CIN processes over successive divisions and assesses the short-term impact of CA formation.^1^

Applying MAGIC to spontaneous micronuclei in near-diploid cells shows that over half of micronucleated MCF10A cells carry at least one de novo CA, a strong enrichment versus normal nuclei, whereas RPE-1 remains largely stable with only occasional CAs. These CAs are dominated by terminal gains and losses, while whole-chromosome alterations are relatively infrequent. Strand-seq analysis of sister-cell pairs reveals dicentric-chromosome–driven breakage–fusion–bridge cycles that generate chains of inverted duplications and can culminate in focal chromothripsis or whole-chromosome pulverisation of a single homologue, positioning dicentrics and self-perpetuating bridges as key drivers of karyotypic diversification even in near-diploid genomes. Using CRISPR-Cas9 to introduce DSBs at defined sub-centromeric, central or sub-telomeric sites, the authors show that break position dictates CA architecture: sub-centromeric cuts generate isochromosomes and stable isodicentrics, whereas central and sub-telomeric cuts yield bridge-mediated inverted duplications and amplified isoacentric fragments that co-segregate in multiples of two. In contrast, transient SAC dysfunction induced by reversine mainly leads to whole-chromosome aneuploidy with few terminal structural CAs, defining a distinct mitotic CIN pattern. Together, these experiments establish MAGIC as a platform to capture spontaneous CIN and dissect CA patterns induced by targeted pre-mitotic and mitotic insults (Fig. 1c).^1^

*TP53* is a key CIN gatekeeper and shapes how often CAs arise and are tolerated across divisions.^1,2,4^ In *TP53*-deficient MCF10A and RPE-1 cells, micronuclei and anaphase bridges accumulate, but unlike in their *TP53*-proficient counterparts, they fail to trigger robust cell cycle arrest, allowing heavily damaged cells to continue cycling. MAGIC combined with single-cell genome profiling shows that *TP53* loss amplifies de novo CA burden and markedly increases complex rearrangements and chromothripsis in otherwise genomically stable RPE-1 cells. Long-term imaging indicates that each mitotic category (normal, lagging, or bridged) generates CAs at similar rates in TP53-proficient and *TP53*-knockout MCF10A cells; what changes is the frequency of aberrant divisions, particularly bridge mitoses, nearly doubling the chance that a given division yields at least one CA.^1^ Alongside other CIN tolerance drivers, such as whole-genome doubling, which buffers the fitness cost of aneuploidy losses, *TP53* inactivation relaxes genome surveillance, hardwiring a persistent supply of new CAs that fuels clonal evolution under therapy while exposing therapeutic vulnerabilities in DNA damage and mitotic surveillance pathways (Fig. 1c).^2,4,5^

Not all newly generated CAs are compatible with continued proliferation.^1,2^ Single-cell cloning shows that micronucleated cells have reduced colony-forming ability compared with their non-micronucleated counterparts, underscoring that many CIN events are acutely deleterious. Among colonies that did emerge, only a fraction of de novo CAs were preserved, indicating strong purifying selection. In MCF10A, losses of 7q, often embedded within complex rearrangements, are disproportionately retained, recapitulating recurrent 7q alterations in breast cancer and suggesting that some CIN-derived lesions confer context-dependent advantages. Across perturbations, MAGIC also reveals a consistent excess of chromosome losses over gains during CA formation, paralleling pan-cancer copy-number landscapes and implying that loss bias is built into CA-generating processes rather than arising solely from negative selection against aneuploid gains.^1,2^ Together with p53-mediated cell cycle arrest and the DNA damage that accumulates in chromatin bridges and micronuclei, this creates a bottleneck in which only certain CA configurations persist: those that maximise tumour fitness by balancing disruptive genome remodelling with sufficient proliferative capacity and pathway-specific advantages and those selected alterations ultimately shape tumour evolution (Fig. 1a, c).^2,4,5^

Taken together, MAGIC provides a high-throughput framework to mechanistically dissect ongoing CIN and reconstruct CA-generating processes across divisions. Within a single experimental setup, Cosenza et al. compared spontaneous CIN with that induced by targeted DSBs or SAC disruption, revealing distinct CA fingerprints. However, CIN trajectories and dominant CA-generating mechanisms are context-dependent, shaped by CIN aetiology, transformation/checkpoint status and tissue of origin, so these conclusions may not generalise across cancers, motivating extension of MAGIC to cancer cell lines and patient-derived organoids. MAGIC is also modular and can be paired with additional upstream perturbations (e.g., chromosome-specific aneuploidies, genotoxic therapies, or drugs targeting pre-mitotic or mitotic processes) and downstream phenotyping and single-cell multi-omics to track CIN in real time (Fig. 1b). Additionally, MAGIC’s phenotype-based enrichment for micronuclei or nuclear atypia may bias inferred CIN trajectories toward nuclear-morphology-visible outcomes, missing events that do not pass through these states or are acutely deleterious and trigger early arrest or cell death. Strand-seq further requires BrdU incorporation during S phase, restricting detection to cycling cells, and has reduced resolution below ~200 kb. Ultimately, MAGIC not only reveals that CIN is “a kind of magic” in tumour evolution but also dissects the tricks behind it, providing a mechanistic basis for understanding how CIN operates.^1^
